# Functional R code is rare in species distribution and abundance papers

**DOI:** 10.1002/ecy.4475

**Published:** 2024-11-20

**Authors:** Kenneth F. Kellner, Jeffrey W. Doser, Jerrold L. Belant

**Affiliations:** ^1^ Department of Fisheries and Wildlife Michigan State University East Lansing Michigan USA; ^2^ Department of Integrative Biology, Ecology, Evolution, and Behavior Program Michigan State University East Lansing Michigan USA; ^3^ Department of Forestry and Environmental Resources North Carolina State University Raleigh North Carolina USA

**Keywords:** abundance, analytic reproducibility, computational reproducibility, distribution, hierarchical models

## Abstract

Analytic reproducibility is important for scientific credibility in ecology, but the extent to which scientific literature meets this criterion is not well understood. We surveyed 497 papers published in 2018–2022 in 9 ecology‐related journals. We focused on papers that used hierarchical models to estimate species distribution and abundance. We determined if papers achieved two components of analytic reproducibility: (1) availability of data and code, and (2) code functionality. We found that 28% of papers made data and code available, and 7% of papers provided code that ran without errors. Our findings indicate that analytic reproducibility remains the exception rather than the rule in ecology literature. We recommend authors (1) test code in a separate clean environment; (2) simplify code structure; (3) minimize software packages used; and (4) minimize code run time. We suggest journals (1) validate authors' provided open data statements and URLs; (2) recommend that code and data be shared in a separate repository rather than as appendices; and (3) elevate the status of code and data during review. We suggest these guidelines can aid the ecology community by improving the scientific reproducibility and credibility of ecological research.

## INTRODUCTION

Scientists are increasingly concerned about reproducibility and replicability of findings across disciplines (Baker, 2016; Open Science Collaboration, 2015), including ecology (Fidler et al., 2017; Nichols et al., 2019). A study's findings are considered *reproducible* if repeating the analysis with the same data yields the same results. A study is *replicable* if a dataset collected independently and under similar conditions yields similar results. Both are crucial to science, but reproducibility is arguably easier to achieve, given that it does not require repeating the experiments or data collection. The ability to accurately and consistently recreate the results of a study, given all the inputs, is sometimes called *computational reproducibility* (Kitzes et al., 2017; Stodden, 2015) or *analytic reproducibility* (LeBel et al., 2018). Analytic reproducibility is important for several reasons. It provides partial evidence of the accuracy of a study's analytics, demonstrating the study's credibility to other scientists (Fišar et al., 2023). Analytic reproducibility also facilitates scientific advancement by ensuring a study's data and analysis specification can be used in later studies and for educational purposes (Jenkins et al., 2023). Hereafter, we will equate analysis specification with analysis code, given the widespread use of the R programing language (R Core Team, 2023) in ecology (Lai et al., 2019). Analysis specification could take other forms such as a detailed written workflow, or screenshots of a software program, which present different challenges for reproducibility.

Though easier than replication, achieving analytic reproducibility is not necessarily easy. An earlier challenge to analytic reproducibility was that the necessary inputs (data, code) were not usually made publicly available or not properly archived. Though sharing data (Bauchner et al., 2016; Stodden et al., 2013; Tenopir et al., 2011) and associated computer code (Culina et al., 2020; Easterbrook, 2014; Stodden et al., 2013; Walters, 2020) is increasingly required by journals for publication, this has not solved the problem. Many papers across disciplines still do not make all required data and code available (Culina et al., 2020; Fišar et al., 2023; Kimmel et al., 2023; Obels et al., 2020; Poongavanan, 2021; Stockemer et al., 2018), though we recognize there can be reasonable constraints on data availability (e.g., locations of endangered species).

Even with data and code made available, challenges remain. For example, the data itself could be the product of processing by software or code that is not provided, limiting reproducibility. Implementing provided code requires technical expertise with the programing language, which not all scientists have, particularly for programing languages less commonly used in ecology like C++, Fortran, or MATLAB. Even with the required technical expertise, reanalyses often do not yield the originally reported results, at least not without corrections and further input from the original authors (Archmiller et al., 2020; Kirouac et al., 2019; Obels et al., 2020; Poongavanan, 2021; Sparks et al., 2023; Stockemer et al., 2018; Stodden et al., 2018; Trisovic et al., 2022).

Given that data, code, and the required technical expertise are available, there are numerous possible reasons for this low reported analytic reproducibility. Perhaps most importantly, there is usually no incentive for authors to ensure their results are analytically reproducible. While journals increasingly require sharing data and code, only rarely is there any external check that, for example, the code runs or matches the reported results (as recommended in Jenkins et al., 2023, but see e.g., Fidler et al., 2017, Fišar et al., 2023). The increasing availability and accessibility of software tools for scientists, such as in the R ecosystem (R Core Team, 2023), has enabled new analyses and techniques. However, this often comes at the cost of increased complexity, and therefore greater potential for problems with the code (Alston & Rick, 2021). Finally, scientists are not always trained in best practices for ensuring their data and code are written and organized in a way that is understandable and usable for others (Hannay et al., 2009; Wilson, 2014).

In summary, we define five components of analytic reproducibility: (1) data availability, (2) data quality/organization, (3) analysis availability, (4) analysis functionality, and (5) ensuring generated output matches published results. In ecology, there is already a substantial literature addressing data availability (Culina et al., 2020; Roche et al., 2015), and there are well‐established guidelines for data quality and organization (Alston & Rick, 2021; British Ecological Society and Cooper, 2017; Jenkins et al., 2023; Wilkinson et al., 2016; Wittman & Aukema, 2020). A few papers have also looked at both data and code availability in ecology; Archmiller et al. (2020) were able to obtain data and code for only 26% of papers from two wildlife journals, Culina et al. (2020) found that only 27% of ecology papers shared data and code, and Poongavanan (2021) found data and code for only 8% of movement ecology papers.

We found only two ecology papers that looked at code functionality and output. Archmiller et al. (2020) looked at two wildlife management journals, finding 68% of papers with data and code available were computationally reproducible. Poongavanan (2021) looked at a single category of analysis (movement), finding 67% of papers had reproducible results. Based on their results, Archmiller et al. (2020) recommend required sharing of data and code, better documentation and metadata, and more training in reproducible workflows. Poongavanan (2021) similarly recommends required sharing data and code, better documentation, and also emphasized the need to record information about the computing environment and software packages used.

Despite these studies, we contend that many ecologists are still largely unaware of analytic reproducibility issues, and do not always prioritize publishing data and code in ways that facilitate reproducibility. Thus, there remains a need to examine the availability and quality of paper code across a larger sample size and a wider range of ecological journals, with the goal of continuing to raise awareness of this problem and identifying specific actionable ways that ecologists can use to improve code functionality.

We addressed this need by assessing two components of analytic reproducibility in a large sample of the ecology literature on species abundance and distribution published during 2018–2022. We selected papers that applied hierarchical models of species abundance and distribution for two reasons. First, understanding species distribution and abundance is a key goal of ecology. Second, hierarchical modeling of species distribution and abundance is our area of expertise, reducing the possibility that unfamiliarity with the statistics used in papers could influence our results (Archmiller et al., 2020). We chose recent papers because journal policies requiring code sharing are relatively recent, and we wanted to maximize the number of papers we found with code available.

We examined two key components of analytic reproducibility:

1. Availability of both data and code. We predicted that:
  a) Papers in journals that had data and code availability policies would be more likely to have both data and code available.
  b) Both data and code availability would increase over time.
2. Code functionality (i.e., does the code run, and if not, why not?). We predicted that:
  a) Greater code complexity (more lines of code, more packages used) would decrease the probability we were able to run the code.
  b) Use of tools designed for reproducibility (R Markdown [Xie et al., 2018], and Docker [Boettiger, 2015]) would increase the probability that code worked.
  c) Code functionality would increase over time, due to increasing awareness of good coding practices and/or higher journal standards.

## METHODS

We preregistered our methodology for this study (Kellner, 2022, 2024). Preregistration requires publicly archiving the study design and/or analysis plans before conducting the study. This guards against questionable research practices such as researchers conducting many analyses and then reporting only the most favorable results (Parker et al., 2016).

### Paper selection

We selected 10 journals that publish studies employing hierarchical modeling to estimate species distribution and abundance. This included ecology, conservation, and wildlife‐specific journals, and so‐called mega journals that publish a wide range of disciplines. The final set of journals was Biological Conservation, Conservation Biology, Ecology, Ecology and Evolution, Ecosphere, Journal of Ecology, Journal of Wildlife Management, Methods in Ecology and Evolution, PLoS One, and Scientific Reports.

Using Web of Science (https://webofknowledge.com), we searched each journal in June 2023 with the following search string: “occupancy OR n‐mixture OR capture recapture OR ‘distance sampling’ OR ‘removal sampling’,” and constrained the results to years 2018–2022. We exported matching records to spreadsheets. For each journal, we randomly sorted the results and reviewed the abstract and main text of each paper. We retained the first 50 papers from each journal that fit our inclusion criteria: (1) research on plants or animals; (2) applied one of the modeling approaches from our search criteria to estimate distribution or abundance for simulated or empirical data; (3) had analyses conducted at least partially using R (R Core Team, 2023).

Many other software packages and programing languages are available for statistical analysis beyond R. We focused on R for two reasons. First, we expected, based on our personal experience, that most papers on species abundance and distribution would use R, or R plus auxiliary software such as JAGS (Plummer, 2003). Our initial sample of papers confirmed this: 80% of the 629 papers that met criteria 1 and 2 above used R, and other work has also found it to be the dominant analysis approach in ecology (Lai et al., 2019). Second, R (as with other programing languages) has the advantage that the analysis steps can be written out explicitly as code, facilitating reproducibility. Conducting this study on papers that used graphical user interface (GUI)‐based software would have been very difficult or impossible without input from the authors.

We chose 50 papers per journal (total sample size = 500). We expected about 150 of 500 papers (30%) to have data and code available (Archmiller et al., 2020; Culina et al., 2020). We chose this sample size based on power analysis and as is typical with power analyses, selected a hypothesized effect size to test a priori. In this study, we tested our power to detect an increase in the probability a paper's code was functional from 0.2 to 0.4. With a sample size of 150, power analysis indicated that we had power >0.8 to detect an effect of this size (Kellner et al., 2024).

### Data and code availability

For each selected paper we recorded the DOI, journal, and year of publication. We then determined if the authors made the dataset, code, or both available online, and if so where they were located (e.g., appendix, database).

### Code functionality

For all papers with data and code available, we downloaded both from the provided source into a unique folder. We did not contact the authors for missing data or code nor try to identify other sources (e.g., unlinked Git repositories). We recorded the format in which the code was provided (script, PDF, R Markdown, or R package), the total number of lines of code, and the number of R packages used, including packages explicitly used and all dependencies.

We attempted to run the code under the following conditions. We used only the data provided with the paper, and did not attempt to run code without the corresponding data. We ignored data and analyses not related to estimation of species distribution or abundance, or that did not use R. We followed any instructions provided in README files. We used the most recent publicly available version of required software, such as R and R packages, unless a certain version was specified by the authors. We recorded as failing to run code that used software no longer available (e.g., R packages removed from CRAN). We excluded papers that used proprietary software from the dataset. We did not make adjustments to the code, even to fix minor issues, except to correctly specify file paths of downloaded data. We recorded if the code ran without errors, and if we encountered an error, we recorded the reason to the best of our knowledge and stopped.

We allocated one hour to run the code from each paper. We made this choice for logistical reasons; with over 100 papers to test, it was infeasible to wait multiple hours or days for each analysis to run completely. This situation was common in our dataset, which included code for extensive simulation studies or Bayesian models with many Markov chain Monte Carlo (MCMC) iterations. In these cases, we attempted to run an abbreviated version (e.g., fewer simulations or iterations) to confirm the code worked and produced output. It is possible that our modifications to the code to change the number of MCMC iterations or simulations could have introduced new errors, or eliminated existing errors. However, we believe these small changes were unlikely to affect code functionality and any introduced changes were likely to cancel each other out (i.e., we were as likely to fix an error as cause one).

### Reproducibility of results

When we planned this study (Kellner, 2022), we intended to assess the third key component of reproducibility: given that the data and code were available and the code runs, do the results obtained match what was reported in the paper? However, we reviewed so few papers for which we were able to completely run the code (due to errors and long run times) that we discarded this part of our study.

### Accuracy check

One author (Kenneth F. Kellner) ran code from all papers and a second author (Jeffrey W. Doser) ran code for a subset (*n* = 30) of papers. We compared results from the two authors to assess accuracy.

### Analysis

We fit two generalized linear mixed models with a logit link, one for each aspect of analytic reproducibility we evaluated: (1) probability a paper had data and code available; and (2) probability a paper's code functioned correctly (ran without error).

#### Data and code availability

The response variable was binary: 1 if a paper had data and code available, and 0 if it did not. Covariates were year and presence of a data and code availability policy for the journal in a given year (binary: 1 = yes, 0 = no), corresponding to our predictions. We obtained data and code policies for each journal in each year, if available, by searching the journal's current web page and past versions of the web page archived on the Wayback Machine (https://web.archive.org/). If the journal only recommended data or code sharing, we did not consider that as a required data or code‐sharing policy. We excluded 58 papers from this model because we were uncertain about the corresponding data and code policy in a particular year based on the archived web pages. During preregistration of this study, we included a prediction for the effects of journal impact factor (IF), but removed this based on reviewer feedback and due to the questionable value of IF for assessing journal quality (Seglen, 1997).

#### Code functionality

The response variable was binary: 1 if the code from a paper ran, 0 if not. Covariates were total lines of code, number of R packages used, code format (PDF/Word, text file, or Rmarkdown/R package), and year. We added the year covariate based on reviewer feedback. We did not compare commented and non‐commented papers as originally planned during preregistration, as 96% of papers included comments in the code and we were unable to objectively determine if comments provided were helpful when running the code.

We included random intercepts by journal for each model. We fit models in a Bayesian framework in R 4.3.1 (R Core Team, 2023) using the rstanarm package (Goodrich et al., 2024). We used default priors (Gabry & Goodrich, 2020) and four chains of 2000 iterations with a warm‐up of 1000 iterations. We assessed posterior convergence visually using traceplots and the R‐hat statistic (<1.1; Vehtari et al., 2021). We assessed model goodness‐of‐fit using posterior predictive checks. Data and code are available on Zenodo (Kellner et al., 2024).

## RESULTS

We identified 500 papers from 9 journals that met our inclusion criteria. Journal of Ecology was excluded from the final dataset because we found only two relevant papers. We added additional papers from the remaining journals to reach 500 total. We excluded three papers written by a co‐author of this study, resulting in a final sample size of 497. The mean number of papers per journal was 55 and the range was 22–75. There were a similar number of papers across years (mean 99, range 80–109).

### Data and code availability

**FIGURE 1:**
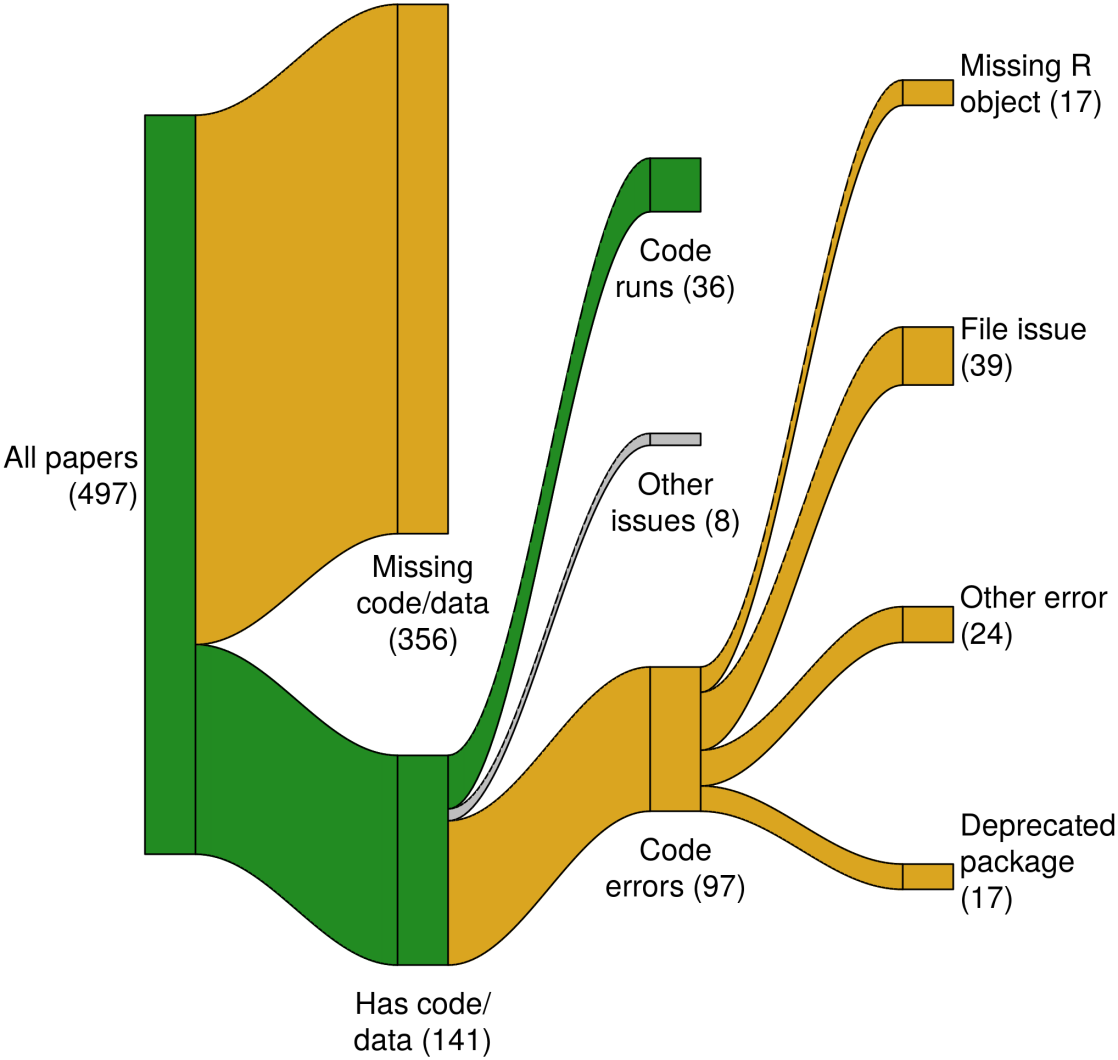
Sankey diagram categorizing data and code availability and code functionality for papers included in analyses. Node labels (and number of papers at each node) are below the associated node. Green indicates a positive outcome (code and data available, code runs) and gold a negative outcome (code unavailable, code does not run). Note that papers could have multiple reasons for why the code didn't run and only the first reason is reflected in this diagram.

Of the 497 papers, 141 (28%) included data and code and were potentially reproducible (Figure 1). Of remaining papers, 109 (22%) had only data, 23 (5%) had only code, and 224 (45%) had neither. Data and/or code were mostly commonly shared using paper appendices (31%), Zenodo (28%), and Dryad (24%). Common data and code sharing problems (excluding not sharing code or data at all) included supplying only BUGS/Stan model code (56 papers), missing or broken links to outside repositories (30 papers), and missing or incomplete appendices referenced in the text (12 papers).

**FIGURE 2:**
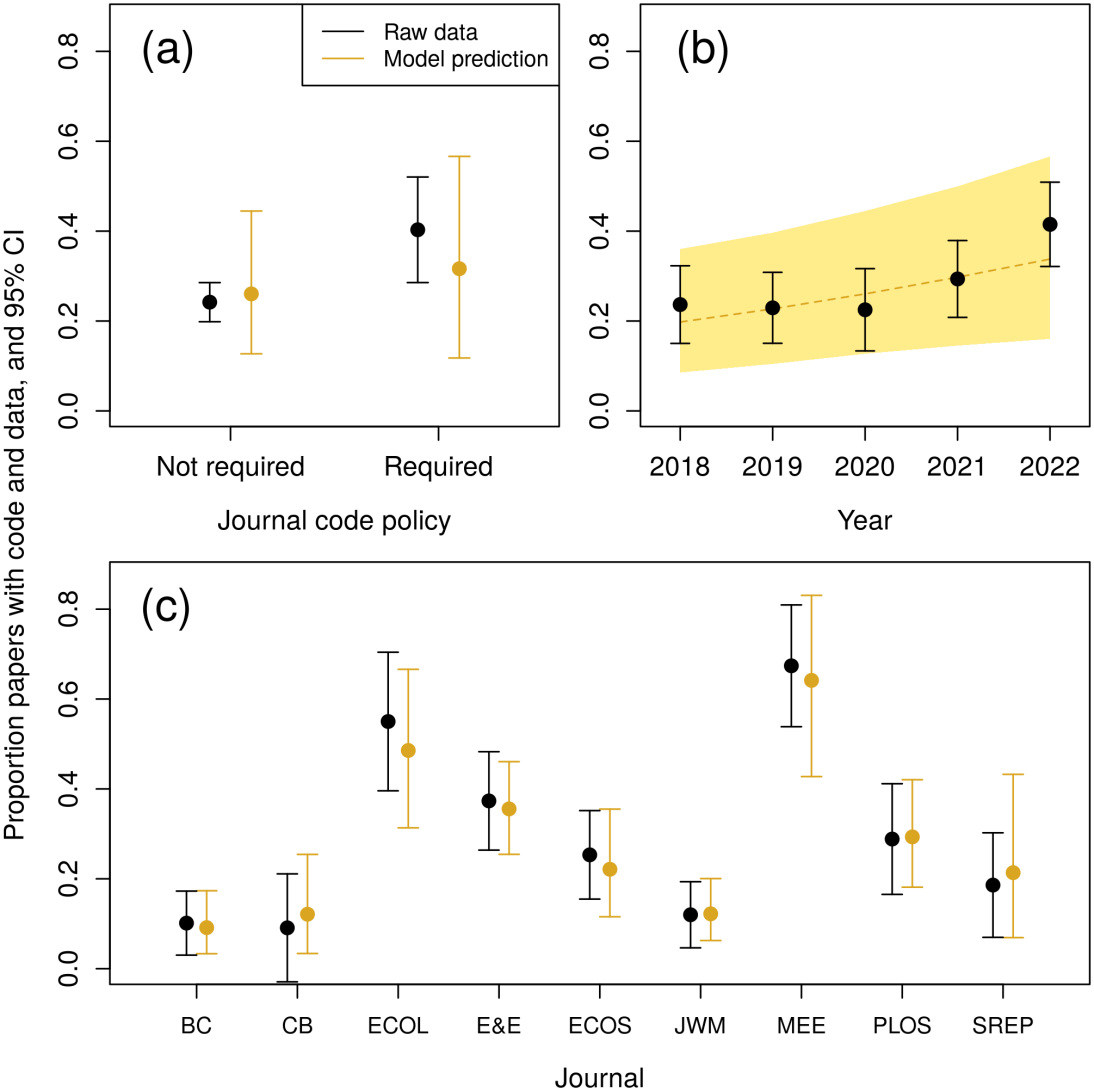
Effects of journal code policy (a), year (b), and journal (c) on proportion of papers that provided complete data and code. Black points and lines represent estimates and 95% CIs based on the raw data, and gold represents predictions (posterior mean and 95% credible interval) from the regression model. In panel c, acronyms correspond to journals as follows: Biological Conservation (BC), Conservation Biology (CB), Ecology (ECOL), Ecology and Evolution (E&E), Ecosphere (ECOS), Journal of Wildlife Management (JWM), Methods in Ecology and Evolution (MEE), PLoS One (PLOS), and Scientific Reports (SREP).

There was a positive effect of year on the probability both data and code were available, with 96% of the posterior samples for the parameter greater than 0. A one‐year increase in publication date corresponded to a 21% (95% credible interval −2% to 49%) increase in the odds a paper had both data and code available (Table 1, Figure 2). The probability a paper had data and code accessible did not differ based on data and code‐sharing policy.

**TABLE 1:** Estimates from the generalized linear mixed model of probability a paper provided data and code, as a function of journal data and code policy and publication year.

Parameter	Estimate	95% credible interval
Intercept	−1.46	−2.37 to −0.57
Code required	0.26	−0.70 to 1.18
Year	0.19	−0.02 to 0.40
Journal random effect SD	1.45	0.40 to 3.80

*Note*: Intercepts were random by journal.

### Code functionality

Of 141 potentially reproducible papers, we excluded 8 (6%) from further analysis due to computer limitations (extremely long runtimes, RAM limitations, etc.). Papers included 1598 ± 2277 (mean ± SD) lines of code and depended on 66 ± 55 R packages. Most papers (119, 89%) provided code primarily in R script format and 9 (7%) used primarily R Markdown or R packages.

We successfully executed all the provided code for 36 of 133 papers (27% of potentially reproducible papers, 7% of all papers; Figure 1). The most common reasons that code failed to run (there could be multiple reasons) were missing or misnamed files (45 papers), reliance on deprecated R packages (17 papers), and missing R objects (17 papers).

**FIGURE 3:**
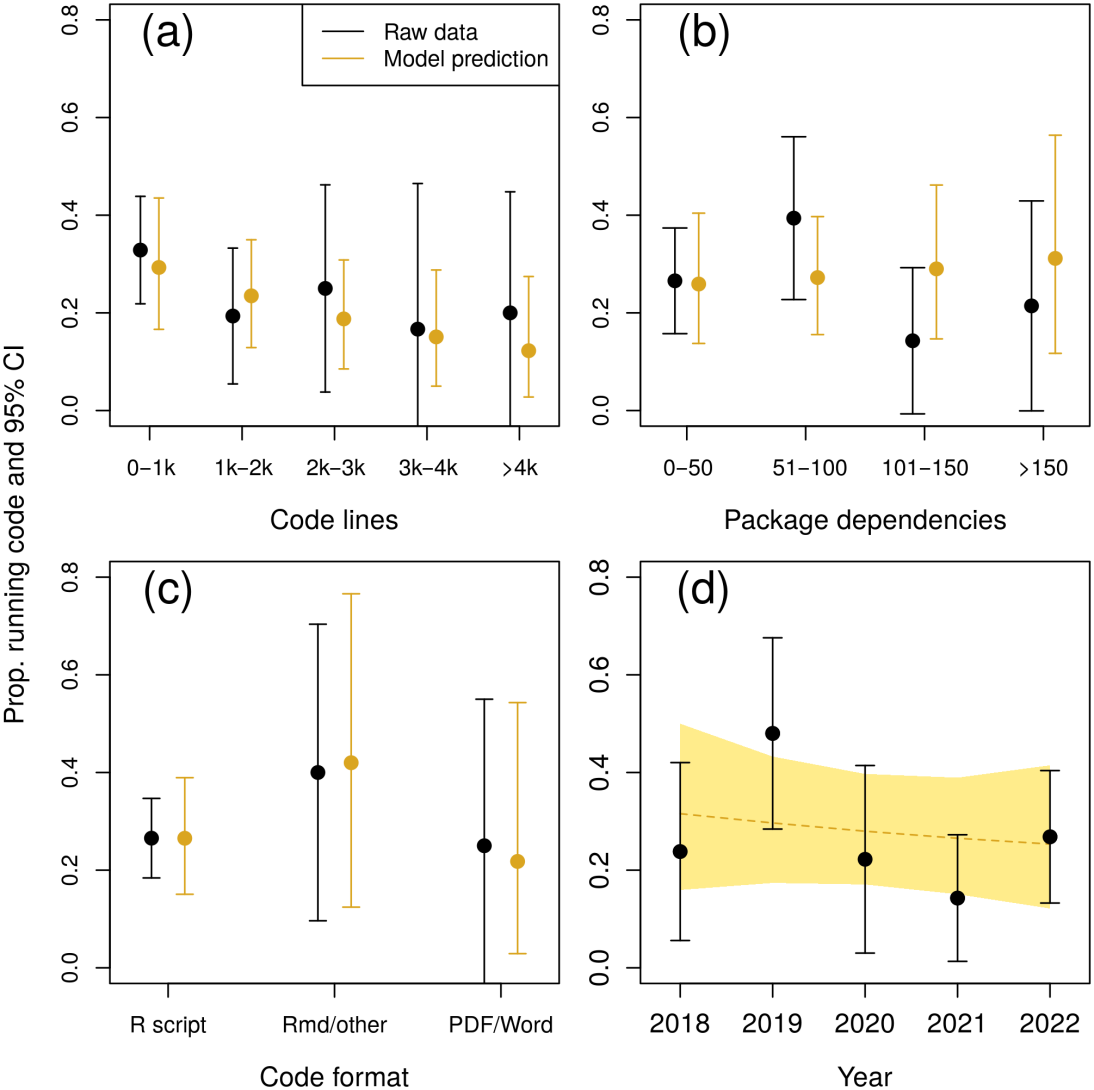
Effects of number of code lines (a), number of required R packages (b), code format (c), and year (d) on proportion of papers for which provided code ran successfully. Black points and lines represent estimates and 95% CIs using the raw data provided, and gold represents predictions (posterior means and 95% credible intervals) from the regression model. In panels (a) and (b), corresponding model predictions were calculated using midpoints of binned data on the *x*‐axis.

The number of lines of code had a negative effect on the probability a paper's code ran successfully, matching our prediction (Table 2, Figure 3). With other covariates held at median or reference levels, a paper with the mean number of lines of code (1605) had a 0.23 probability of the code running, while a paper with lines of code one SD above the mean (3867) had a 0.13 probability of the code running. The probability that a paper's code ran successfully was not related to the number of packages used, code format, or year.

**TABLE 2:** Estimates from the generalized linear mixed model of probability a paper's code ran successfully, as a function of number of lines of code, number of R packages required, code format (relative to a reference level of R script), and year.

Parameter	Estimate	95% credible interval
Intercept	−0.98	−1.89 to −0.14
Code lines	−0.69	−1.53 to −0.01
Packages	0.09	−0.41 to 0.57
Rmd/R package	0.67	−0.82 to 2.20
PDF/Word	−0.45	−2.48 to 1.27
Year	−0.08	−0.37 to 0.21
Journal random effect SD	0.22	0.00 to 1.16

*Note*: Intercepts were random by journal.

### Accuracy check

Of the 30 papers for which two authors executed code, we obtained the same outcome for 28 papers (93%). One paper was a false positive (error reported when code actually ran) and one was a false negative (code reported running but actually had an error). These papers were corrected in the final dataset used for analysis. One additional disagreement was likely due to differences in computing environment and was ignored.

## DISCUSSION

The state of data and code sharing in the ecology literature we examined is concerning. Only 28% of papers shared enough information to potentially be reproducible, although this percentage increased over time. Furthermore, only 27% of papers with data and code available (7% of all papers) provided code that executed successfully. We recommend analytic reproducibility should be a base criterion for scientific credibility (Fišar et al., 2023), so it is alarming that >90% of papers we reviewed failed to meet this criterion.

### Data and code availability

Our results are similar to Archmiller et al. (2020) and Culina et al. (2020), who found that 26% and 27% of papers, respectively, shared data and code. In contrast to Culina et al. (2020), we found some evidence that this percentage has increased over time. Culina et al. (2020) assessed papers during 2015–2019, whereas, we assessed papers from 2018 to 2022, so our findings may reflect updated journal requirements for data and code sharing. However, in contrast to our prediction, we did not find evidence of an effect of journal policies requiring data and code on the probability a paper shared data and code. Even strict data and code‐sharing requirements typically have exceptions, such as for threatened or endangered species, which are likely particularly relevant for studies of species distributions. Furthermore, we identified many papers (*n* = 49, 10%) that claimed to share data and code we were unable to access due to broken URLs and missing appendices.

The percentage of papers that shared data and code varied greatly among the nine journals we examined. The journals *Ecology* and *Methods in Ecology and Evolution* had comparatively high levels of code sharing (55% and 67% percent of papers, respectively, in 2018–2022). Both currently have relatively stringent data and code‐sharing guidelines (Ecology, 2024; Methods in Ecology and Evolution, 2024), and *Methods in Ecology and Evolution* makes code review an explicit part of the review process (Methods in Ecology and Evolution, 2024). The lower availability of data and code for *Conservation Biology* and *Biological Conservation* could reflect, in part, an emphasis on studies of threatened and endangered species for which data often cannot be shared.

### Code functionality

Problems executing shared code were remarkably common. Overall, we found evidence for our prediction that the more lines of code there were (as a crude measure of complexity), the lower the chance it ran successfully. Many problems we encountered were relatively minor, such as misnamed files (45 of 141 papers) and missing R objects (17 of 141 papers). With some professional judgment from the person running the code or input from the original authors, these minor problems should be easy to resolve. However, their presence indicates authors do not always thoroughly test their shared code before submission. We suggest authors take these minor errors seriously as even simple changes that future scientists (including the authors themselves) are required to make to get the code running can introduce ambiguity into the process and results. Of course, minor mistakes in the code can also mask more serious underlying issues. We did not attempt to test this (e.g., by repeatedly fixing issues we found and rerunning the code).

Another common reason that code failed to run was problems with the external R packages used in the code (17 of 141 papers, 12%). Most of these were due to the recent deprecation and removal from CRAN of a suite of R packages for processing spatial data widely used by ecologists (notably the packages rgdal and maptools; Pebesma & Bivand, 2023). While problems with deprecated packages are not due to paper authors, this issue does raise concerns about the degree to which shared data and code depend on R packages typically maintained by volunteers. We found that code from the average paper in our dataset depended directly or indirectly on 66 R packages. A future deprecation or bug for any of these packages could potentially break the shared code, or (possibly worse) yield meaningfully different results. For example, a recent widely cited paper claiming a reduction in scientific disruption over time (Park et al., 2023) reported results that may be undermined by a bug in a Python plotting package (Holst et al., 2024). However, in contrast to our prediction, the number of packages used did not affect the probability a paper's code ran successfully. Given that we considered only recent papers (2018–2022), we would expect to see a stronger negative relationship as code from these papers ages and additional package changes and deprecations increase.

The proliferation of specialized R packages has given ecologists easy access to a vast range of analysis tools, but this comes at the cost of an increasingly complex and fragile network of code dependencies that can, and likely will, be broken in the future. We think this is a trade‐off that should be carefully considered by ecologists. When possible, ecologists and package developers should prioritize using fewer packages with fewer dependencies, reducing the chances of future code issues. Additionally, there are tools such as Renv (Ushey et al., 2024), groundhog (Simonsohn & Gruson, 2024), and Docker (Boettiger, 2015) which can be used to reliably recreate the exact set and versions of packages used. We used Docker and Rocker (Boettiger & Eddelbuettel, 2017) in the analysis for this paper to set R to version 4.3.1 and R packages to the versions available on 30 October 2023 (Kellner et al., 2024). In March 2024, rerunning the analysis with the latest R and R package versions already yielded slightly different numeric results, emphasizing the importance of recording the package versions used.

Finally, we note that our assessment of code functionality required correctly specified code and appropriately organized and formatted data. For some papers where the code failed to function, it is possible that the code itself was correct, and errors were actually due to problems with the data. Though we did not attempt to identify these issues, in these cases, the code itself could be valuable for applications in other studies.

### Recommendations for authors

We suggest that all authors of papers (not just the authors conducting the analysis) should review the data and analysis code with the following recommendations in mind. See Ivimey‐Cook et al. (2023) for guidelines on reviewing code.

1. Authors should test their code in a separate, clean environment before code archival. This could involve explicitly clearing the R environment before running the code (e.g., with rm.(list = ls())), or requesting a colleague or coauthor to run the code on their computer. Such a test will reduce or eliminate two of the most common code problems we found (missing files and R objects).
2. Keep the shared data and code structure as simple as possible. Other articles have described suggested file naming schemes and folder structures (Alston & Rick, 2021; British Ecological Society and Cooper, 2017). We will not repeat these helpful guidelines, but emphasize that we found the easiest analyses to reproduce contained a single folder and a single main code file, removing ambiguity in the order in which things should be run. Tools like R Markdown are also a good option, although we did not find evidence that use of R Markdown had any effect on the probability code ran successfully.
3. Think critically about the software packages you depend on. One of the three largest issues we found that impacted code functionality was unavailability of R packages required in the code. Package availability and maintenance is largely dependent on volunteer effort, and there is no guarantee packages we use now will be available in the future. A single broken or missing package can render code non‐functional. Consider whether you need to use a powerful but many‐dependency package, or if you can use the simpler tools included with base R. Record the versions of each package you used (Poongavanan, 2021). Ideally, use a tool like Renv (Ushey et al., 2024), groundhog (Simonsohn & Gruson, 2024), or Docker (Boettiger, 2015) that can recreate the specific version of packages used in an analysis to reduce future package changes or deprecations.
4. Recognize the costs of code run time. Very long run times can prevent you and other scientists from checking that your code reliably reproduces your results. Consider faster alternatives (such as using maximum likelihood instead of a Bayesian approach) if they are otherwise equivalent. At minimum, provide in your code test settings that allow other scientists to quickly confirm that your code runs.

### Recommendations for journals

1. Check authors' open data statements and provided DOIs and URLs carefully. In numerous cases authors stated they shared the data/code but did not, or provided DOIs or URLs that were broken.
2. Recommend to authors that code and data be shared in a separate repository, instead of as appendices. This makes the data and code directly citable. This also prevents a common problem we found where journals scrambled the names of uploaded files in appendices using a systematic renaming scheme, meaning the code referenced names of data files or other code files that were now “missing.” If data and code must be uploaded as an appendix, we recommend that journals require it to be in a compressed folder to avoid this problem.
3. Consider further elevating the status of data and code in the review process. This could take the form of sharing the data and code with reviewers to be reviewed along with the manuscript (Ivimey‐Cook et al., 2023; Methods in Ecology and Evolution, 2024) or a data editor that independently checks data and code packages (American Journal of Political Science, 2024; Bolnick, 2022; Ecology Letters, 2024).

## CONCLUSION

Ecology, like many scientific disciplines, increasingly recognizes the importance of reproducibility and replicability in research. Sharing data and code that can accurately recreate the results of a scientific study is a necessary component of reproducible research. Our review of 497 papers in 9 ecology journals highlights that functional code is the exception rather than the norm in ecology. We provide a series of recommendations to both authors and journals to improve the state of analytic reproducibility in ecology. We believe our guidelines will assist the ecology community in improving the scientific credibility of ecological research.

## AUTHOR CONTRIBUTIONS

All authors contributed critically to the drafts and gave final approval for publication. Kenneth F. Kellner and Jerrold L. Belant designed the study. Kenneth F. Kellner and Jeffrey W. Doser collected data. Kenneth F. Kellner analyzed data and led the writing of the manuscript.

## FUNDING INFORMATION

Partial support was provided by the Boone and Crockett Program in Wildlife Conservation at Michigan State University.

## CONFLICT OF INTEREST STATEMENT

The authors declare no conflicts of interest.

## Data Availability

Data and code (Kellner et al., 2024) are available in Zenodo at https://doi.org/10.5281/zenodo.13940818.
